# Optimizing conditions for labeling of mesenchymal stromal cells (MSCs) with gold nanoparticles: a prerequisite for in vivo tracking of MSCs

**DOI:** 10.1186/s12951-017-0258-5

**Published:** 2017-03-29

**Authors:** Philipp Nold, Raimo Hartmann, Neus Feliu, Karsten Kantner, Mahmoud Gamal, Beatriz Pelaz, Jonas Hühn, Xing Sun, Philipp Jungebluth, Pablo del Pino, Holger Hackstein, Paolo Macchiarini, Wolfgang J. Parak, Cornelia Brendel

**Affiliations:** 1 Department of Hematology, Oncology and Immunology, Philipps University Marburg, Marburg, Germany; 2 Department of Physics, Philipps-University of Marburg, Marburg, Germany; 3 Thoraxklinik at Heidelberg University Hospital, Heidelberg, Germany; 4 Institute for Clinical Immunology and Transfusion Medicine, Justus-Liebig University Giessen, Giessen, Germany; 5 Laboratory of Bioengineering & Regenerative Medicine (BioReM), Kazan Federal University, Kazan, Russia; 6 CIC Biomagune, San Sebastián, Spain

**Keywords:** Mesenchymal stromal cells (MSCs), Au nanoparticles (Au NP), labeling, in vivo tracking

## Abstract

**Background:**

Mesenchymal stromal cells (MSCs) have an inherent migratory capacity towards tumor tissue in vivo. With the future objective to quantify the tumor homing efficacy of MSCs, as first step in this direction we investigated the use of inorganic nanoparticles (NPs), in particular ca. 4 nm-sized Au NPs, for MSC labeling. Time dependent uptake efficiencies of NPs at different exposure concentrations and times were determined via inductively coupled plasma mass spectrometry (ICP-MS).

**Results:**

The labeling efficiency of the MSCs was determined in terms of the amount of exocytosed NPs versus the amount of initially endocytosed NPs, demonstrating that at high concentrations the internalized Au NPs were exocytosed over time, leading to continuous exhaustion. While exposure to NPs did not significantly impair cell viability or expression of surface markers, even at high dose levels, MSCs were significantly affected in their proliferation and migration potential. These results demonstrate that proliferation or migration assays are more suitable to evaluate whether labeling of MSCs with certain amounts of NPs exerts distress on cells. However, despite optimized conditions the labeling efficiency varied considerably in MSC lots from different donors, indicating cell specific loading capacities for NPs. Finally, we determined the detection limits of Au NP-labeled MSCs within murine tissue employing ICP-MS and demonstrate the distribution and homing of NP labeled MSCs in vivo.

**Conclusion:**

Although large amounts of NPs improve contrast for imaging, duration and extend of labeling needs to be adjusted carefully to avoid functional deficits in MSCs. We established an optimized labeling strategy for human MSCs with Au NPs that preserves their migratory capacity in vivo.

**Electronic supplementary material:**

The online version of this article (doi:10.1186/s12951-017-0258-5) contains supplementary material, which is available to authorized users.

## Background

Mesenchymal stromal cells (MSCs) exhibit a high ex vivo expansion capacity and have already entered the clinic as cellular products for various applications [1, 2]. They possess anti-inflammatory and regenerative potential, and migrate into sites of inflammation, tissue repair, and neoplasia [3–5]. Due to their properties and safety, they are considered as a promising tool in regenerative medicine and oncology. About 200 clinical phase I/II and III studies revealed no side effects, even in allogenic settings [6]. In oncology, new therapeutic concepts envision e.g. genetically modified MSCs as a vehicle to selectively deliver anti-tumorigenic proteins or compounds to neoplastic tissue [7]. The efficacy of these approaches, as well as the extent of side effects, is directly linked to the potential of MSCs to accumulate in tumors after systemic administration. In the context of regenerative medicine, MSCs are used a promising therapeutic approach to repopulate extracellular matrixes, with the function to repair and reconstruct complex tissues. Thus, the clinical use of MSCs has overcome its infancy steps [8]. Still, many details remain to be unraveled. This involves for example the mechanisms of homing, and in particular also the in vivo fate of MSCs. This circumstance evokes the necessity for a noninvasive in vivo MSC tracking method that does not influence their biological properties and cellular function, is highly specific to the target cells, is biocompatible, safe and nontoxic, and allows for quantification of low MSC numbers in invaded tissue [9]. Stem cell-tracking methods being currently used rely on labeling the cells with fluorescent molecules for optical imaging, radionuclides for positron or gamma photon emission tomography (PET), or labeling with certain contrast agents, such us exogenous elements, which either allow visualization by magnetic resonance imaging (MRI) or can be detected by mass spectrometry [10–15]. In the first case the application is limited to small animals or intraoperative use, due to light adsorption in thick tissue. The second approach requires extensive preparation of MSCs and handling of radioactive materials. In the third case, when using mass spectrometry as detection method, tissue decomposition prior to measurements is needed. All methods are limited in sensitivity by non-sufficient cell labeling efficiency, or require extensive tissue treatment for further imaging and detection. Combined with low stem cell homing, efficiency revised labeling considerations are needed.

Non-invasive imaging of MSCs after labeling with inorganic colloidal nanoparticles (NPs) is a promising tool that allows for recording distributions and the long-term tracking of the MSCs after systemic application {Huang, 2014 #32200; Skopalik, 2014 #32201; Schmidtke-Schrezenmeier, 2011 #32202; Betzer, 2015 #32883; Meir, 2015 #32885}. In comparison to organic molecules, inorganic NPs may allow for higher contrast in certain imaging techniques, such as magnetic resonance imaging (MRI) and computer tomography (CT). In MRI, FeO_x_ NPs have been demonstrated to provide good contrast in transverse relaxation time (T_2_)-based imaging [16, 17]. In CT, the best contrast is obtained for elements with high atomic number. Thus, Au NPs are good candidates for labeling strategies [18, 19]. For our study we employed 4.25 (±0.88) nm Au NPs coated with the amphiphilic polymer poly (isobutylene-*alt*-maleic anhydride) modified with dodecylamine (PMA). The NPs were purified via gel electrophoresis or ultracentrifugation, and subjected to full characterization as previously reported [20]. This included UV/Vis absorption spectroscopy, transmission electron microscopy (TEM), and dynamic light scattering (DLS). NPs are in general readily endocytosed by cells [21, 22], and thus, labeling of MSCs in principle is straightforward. Upon cell division, the NPs are passed to the two daughter cells [23].

However, while in principle, the concept seams easily to be conducted, labeling of MSCs with NPs has to be performed under a delicate balance. From the imaging point of view, more NPs inside each MSC would relate directly to better contrast in imaging. On the other hand, it is reasonable to reduce the amount of NPs inside each MSC as much as possible, in order to avoid potential cytotoxic effects. Thus, labeling conditions need to be carefully optimized. Au NPs are promising candidates, as their biocompatibility at low doses is well-accepted [24]. Gold has been used for example as clinical therapeutic in patients with severe rheumatologic disorder for many years, with well-known safety profit and limited side effects [25]. Gold is usually not present in living organisms and thus, tracing of Au NPs by mass spectrometry benefits from low background signals, in contrast to FeO_x_ NPs, as there is a significant level of endogenous/constitutive iron. Recent studies have shown that Au NPs at least partially fulfill basic requirements for efficient long term labeling of MSCs, i.e. long term stability, low cytotoxicity, and most importantly, no interference with cellular functioning. Ricles et al. have demonstrated that lysine coated Au NPs of hydrodynamic diameters of around 50 nm do not interfere with differentiation [26]. Long term tracking for a period of 2 weeks seems feasible, due to high retention times and low cytotoxicity. In contrast with these findings, some studies revealed a negative effect of Au NPs on certain cellular functions such as proliferation [27, 28]. In addition the morphology of subcellular structures seems to be disturbed depending on the applied dose [29].

To further assess the biocompatibility and suitability of Au NPs for MSC tracking, we investigated cellular responses to Au NP labeling in MSC derived humans (hMSCs), such as uptake, cytotoxicity, proliferation, migration, morphology, immunophenotype, and in vivo biodistribution. For MSC detection via mass spectrometry we elucidated the detection sensitivity by quantifying the required number of labeled cells to be able to prove MSC presence in a population of cancer cells.

## Results

### Au NPs are readily incorporated by MSCs

**Fig. 1:**
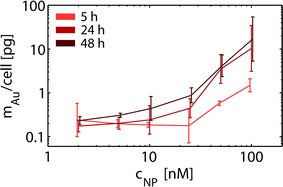
Quantitative determination of uptake of Au NPs by MSCs. hMSCs were incubated with Au NPs (ca. 4 nm core diameter) with different concentrations (c_NP_ = 2–100 nM) for a range of times (5, 24, 48 h). After washing, the mass m_Au_ of intracellular Au was determined by ICP-MS and was normalized to the initial number of cells. Results are presented as mean value ± relative error (derived from propagation of standard deviation (SD) from at least three independent experiments using cells from different donors

**Fig. 2:**
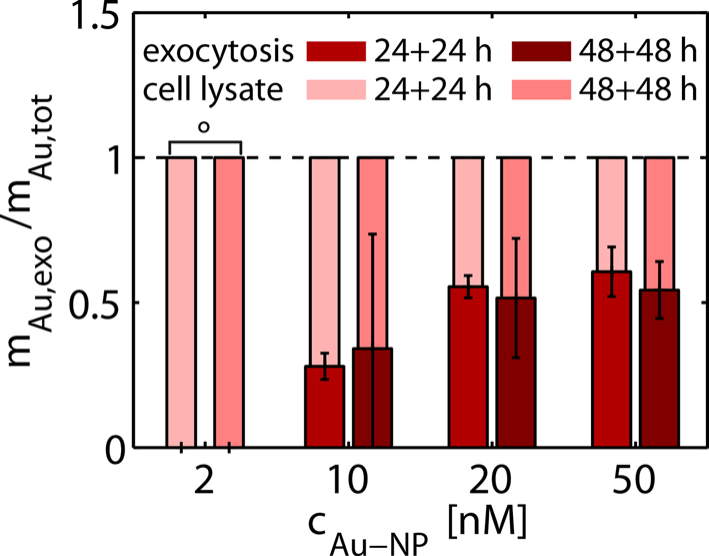
Endocytic uptake and exocytosis of Au NPs by MSCs. MSCs were exposed to Au NPs (ca. 4 nm core diameter) at the indicated doses c_NP_ for 24 or 48 h. After washing off residual NPs from the medium and the outer cell membrane, i.e. NPs which had not been internalized by the MSCs, culturing was continued in fresh growth medium for 24 or 48 h. Then, the amount of intracellular Au NP m_Au_ (i.e. amount of elemental Au inside the cell pellet) and exocytozed Au NPs (i.e. amount of elemental Au in the medium) m_Au,exo_ were determined by ICP-MS. Finally, the fraction of exocytosed Au NPs was determined as m_Au,exo_/(m_Au,exo_ + m_Au_) = m_Au,exo_/m_Au,tot_. For cells labeled with c_NP_ = 2 nM (°) the Au content in the cell medium was below the detection limit

We monitored the incorporation of Au NPs into MSCs in a dose- and time-dependent manner, cf. Fig. 1. In the present study, ca. 4 nm core-sized Au NPs and exposure concentrations ranging from c_NP_ = 1 to 100 nM were used [30]. The uptake was quantified by determination of elemental Au levels inside cells via ICP-MS (cf. Fig. 1). In general the amount of internalized NPs increased over time, whereby after long exposure times (>24 h) and high NP concentrations saturation effects could be observed, cf. Fig. 1. The data shown in Fig. 1 allow for calculating the average number N_NP_ of Au NPs, which were internalized by each cell as N_NP_ = (m_Au_/M_Au_)·N_A_, with m_Au_ being the mass of elemental Au inside each cell as detected with ICP-MS (cf. Fig. 1), M_Au_ = 196 g/mol the molar mass of Au, and Avogadro’s constant N_A_ = 6.02·10^23^/mol. In case of exposure to c_NP_ = 10 nM Au NPs for 24 h this results in N_NP_ ≈ 4·10^5^ NPs per cell, approximating each NP as a sphere of core diameter d_c_ = 4.2 nm (i.e. ca. 4 nm) and constant density ignoring the extent in volume due to the polymer coating. For comparison, at c_NP_ = 10 nM around 6·10^12^ NPs are contained in 1 mL of growth medium. In fact, only a small fraction of NPs present in the medium was actually incorporated by cells, as known also from previous studies [31]. The classical uptake pathway of NPs by cells is endocytosis [22], and internalized NPs are enriched in intracellular vesicles. Therefore, NP excretion was investigated by measuring the increasing Au content in the extracellular medium 24 or 48 h after labeling, as depicted in Fig. 2. The data demonstrate that with rising NP concentrations the excretion via exocytosis is also increasing, which takes place largely within the first 24 h. Note that ICP-MS can not distinguish between Au NPs just adherent to the outer cell membrane and Au NPs that have been in fact endocytosed. There are methods available which allow for separating both populations [32, 33]. However, we did not apply this analysis, as it would not be relevant for the in vivo studies, as discussed in the respective paragraph.

### Functional impact of Au NP labeling on MSCs

**Fig. 3:**
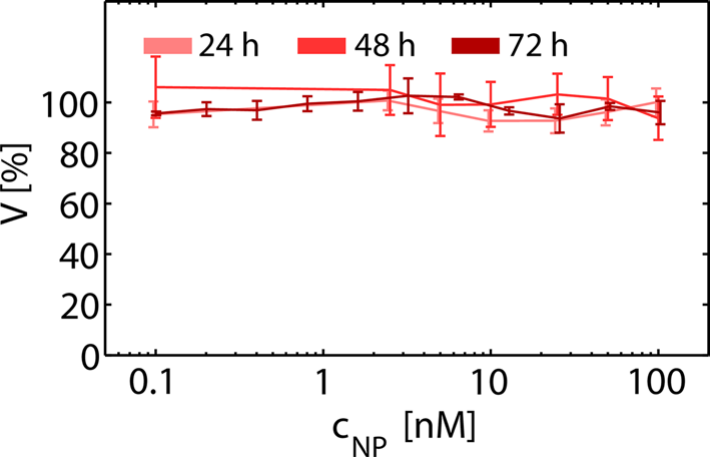
Cell viability of MCS exposed to ca. 4 nm core diameter Au NPs. Cell viability of MSCs following exposure of Au NPs. MSCs were exposed to various concentrations c_NP_ of Au NPs at different incubation times (24–72 h). The cell viabilities were normalized to the viability of cells, which had not been exposed to NPs (control media). Results are presented as mean value ± SD from three independent experiments using cells from different donors

**Fig. 4:**
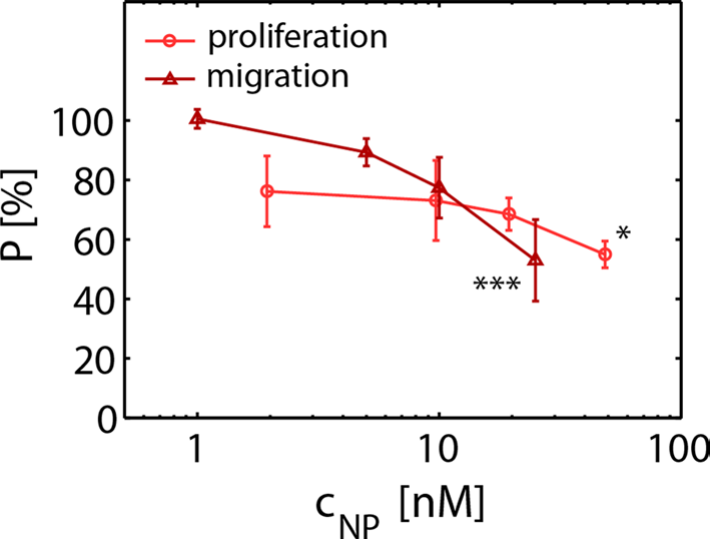
Effect of Au NP exposure on MSC in vitro proliferation and migration. The proliferation potential P upon exposure to Au NPs was normalized to that of untreated cells (c_NP_ = 0 nM) and those treated with a mitosis inhibitor. Proliferation of MSCs exposed to c_NP_ = 50 nM (24 h exposure) was significantly reduced (p < 0.05). The migration capacity after Au NP labeling is displayed as the ratio of the number of migrated cells N_mig_ divided by the total cell number, which is the sum of non-migrated cells N_non–mig_ and migrated cells: N_tot_ = N_mig_ + N_non–mig_. The results were normalized to that of untreated cells (c_NP_ = 0 nM) and to the negative control, where serum free media was used in the lower compartment. Migration of MSCs exposed to c_NP_ = 25 nM was significantly reduced (p < 0.001)

**Fig. 5:**
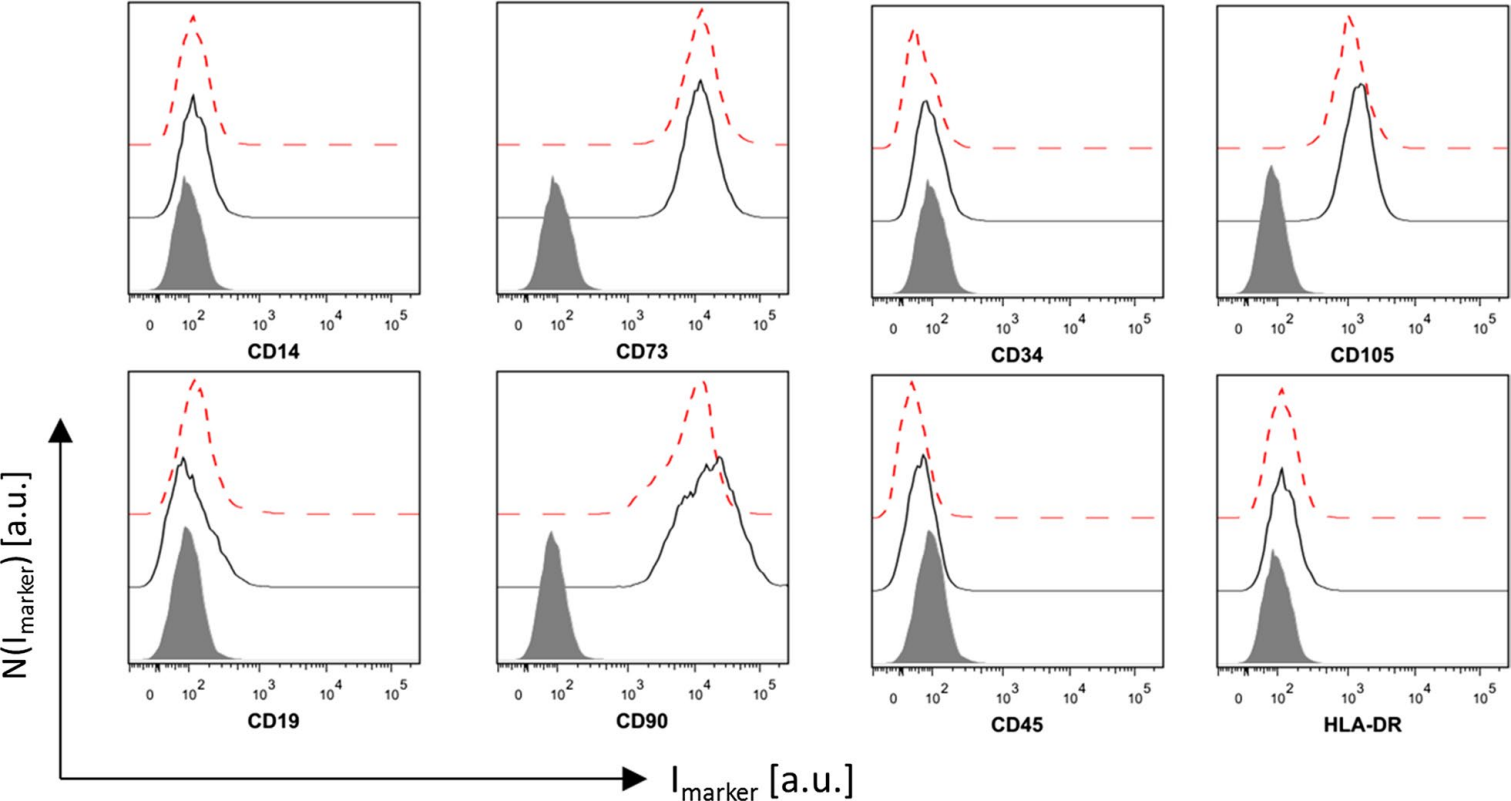
Internalized Au NPs do not affect surface marker expression of MSCs. Representative histograms of 3 independent experiments of the distribution of the marker fluorescence N(I_marker_) of MSC-defining surface markers of untreated MSCs (*black solid line*) and MSCs exposed to Au NPs at c_NP_ = 10 nM (*red dashed line*) for 48 h are shown. The *solid grey front curve* represents the isotype control

These observations prompted us to investigate the biocompatibility of the ca. 4 nm Au NPs. Cell viability after exposure to Au NPs was assessed employing the resazurin (AlamarBlue) assay [34, 35]. The data shown in Fig. 3 indicate that the cell viability of human MSCs exposed to NPs for 24 and 48 h was not strongly affected. However, a trend for a decrease of cell viability was observed at high NP concentrations (c_NP_ > 50 nM) at longer exposure times (72 h). Because cell viability as measured by the resazurin assay has limited sensitivity as indicator for probing effects of NPs on MSCs, we additionally carried out a NP-concentration dependent proliferation assay, cf. Fig. 4. Relative cellular proliferation was significantly reduced for c_NP_ = 50 nM. In fact already at very low NP doses of 2 nM, there is a tendency of concentration-dependent reduction of proliferation. Moreover, migratory functioning is of particular importance for in vivo homing of MSC in tumor tissue. In several studies MSCs were used for homing and tracking experiments [36]. As depicted in Fig. 4 we demonstrate that cellular migration through a porous membrane [37] was affected in case cells have incorporated NPs. Our data suggest a dose dependent inhibitory effect on the migration capacity of MSCs labeled with Au NPs. A significant negative effect was already visible for c_NP_ = 50 nM. Based on our data, we identified the least tolerable dose of ca. 4 nm diameter Au NPs exposed to MSC for 48 h to be around 10 nM. In order to probe whether labeling of MSCs under these conditions affects the immunophenotype of MSCs, expression of surface markers was determined by flow cytometric analysis upon exposure to NPs. Our results showed that Au NP-labeled MSCs maintained their characteristic immunophenotype, as determined by expression analysis of CD73, CD90 and CD105. MSC did not express CD14, CD19, CD34, CD45, and HLA-DR as shown in Fig. 5. The immunophenotype of MSCs labeled under these conditions is in accordance with the consensus criteria [38]. This is in line with results obtained by Mailänder et al., who showed no impact on lineage markers and differentiation [39] upon NP-labeling of MSCs. In this way, at reasonable Au NP concentrations (i.e. 10 nM for ca. 4 nm Au NPs), the NP labeling does not interfere with the immunophenotype, and does not cause long-term cytotoxicity. However, our data reveal onset of negative effects on proliferation and migration potential already at these concentrations. Taken together the amount of Au NPs which can be reasonably added as label per cell is clearly limited, affecting the maximum contrast which can be obtained.

#### The Au NP labeling capacity of MSC is donor dependent

**Fig. 6:**
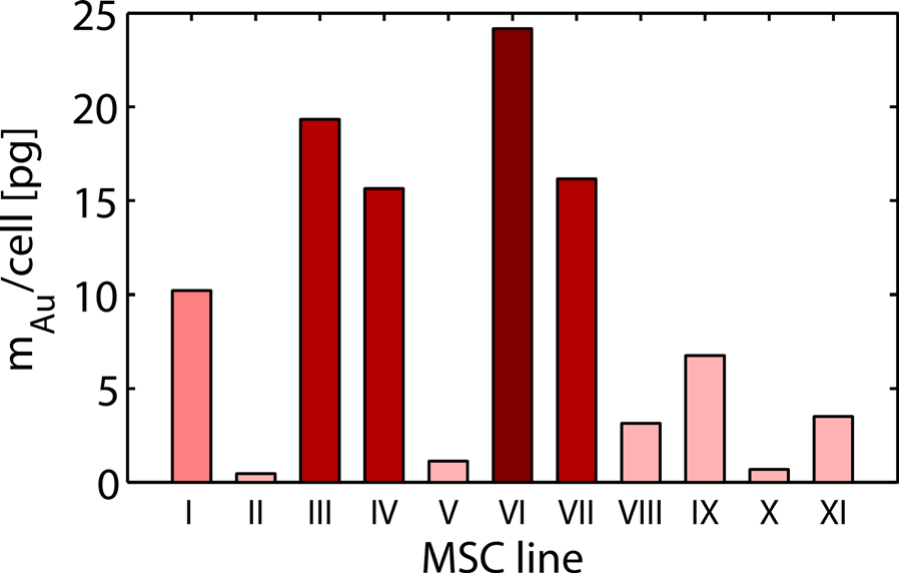
Au NP uptake capacity is donor dependent. MSCs in 3rd or 4th passage were labeled with 10 nM Au NPs for 48 h in vitro. Internalized Au NPs were measured by ICP-MS, revealing considerable variations of Au NP uptake between eleven different MSC donors. Experiments were performed independent to preceding measurements. A different batch of Au NPs was used, which was purified by ultracentrifugation instead by gel electrophoresis. Thus, absolute Au NP content as compared to Fig. 1 may vary

In order to determine the efficacy of MSC labeling with Au NPs at an optimized concentration of 10 nM, MSCs from eleven different donors were incubated with Au NP for 48 h. MSCs were all in passage 3 to 4, because many cell doublings may impair cell functioning and differentiation [40]. Although the same optimized labeling strategy and Au NP concentration was applied, uptake of Au NPs varied considerably in MSCs derived from different individuals as shown in Fig. 6. Thus, other parameters apart from size or concentration of Au NPs must be responsible for the biologic variation in NP tolerance of MSCs, and testing of labeling efficiencies is mandatory for subsequent in vivo tracking experiments with NP labeled MSCs.

#### Linear MSC detection mode with ICP-MS

**Fig. 7:**
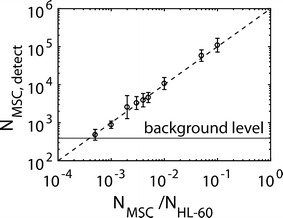
Linear dose dependency of MSC detection employing ICP-MS. N_MSC_ = 0–100,000 MSCs labeled with c_NP_ = 10 nM of Au NPs for 48 h were mixed with N_HL_ = 10^6^ unlabeled HL-60 cells. Then, the number N_MSC,detect_ of MSCs in the mixture was determined via ICP-MS. The dashed line represents the expected results under optimal conditions. The Au noise background level and therefore the detection limit was corresponding to N_MSC,limit_ ~ 400 labeled cells

Using our optimized parameters for NP-labeling of MSCs, we sought to determine whether MSC detection with ICP-MS follows a linear dose response relation. ICP-MS is a frequently used tool for determining bio-distributions, in particular of Au NPs. Upon homing, MSCs actually will only form a small fraction of cells on the target site. For a limiting dilution assay approach we were able to detect as little as 400 labeled MSCs (c_NP_ = 10 nM, 24 h) within a population of 10^6^ acute myeloid leukemia cells (HL-60). Thus, cell numbers down to 400 labeled MSCs/10^6^ HL-60 cells are resolvable, before signal cannot be discriminated from the background any more. This corresponds to as little as 0.04% cells. The ratio between expected numbers of labeled cell under optimal conditions and detected MSC via ICP-MSC was linear (cf. Fig. 7). This allows for some estimation about the minimum tissue volume V_min_ which could be detected via homing of MSCs. In case one assumes a mean value V_cell_ for the volume of one cell, the tissue volume which can be resolved would be V_min_ = V_cell_·N_MSC,limit_/(N_MSC_/N_cell_). Using the experimentally determined value N_MSC,limit_ ≈ 400 (cf. Fig. 7) and the numbers N_MSC_/N_cell_ = 10^−5^ and V_cell_ = 100–1000 μm^3^ [41] as example, the smallest structure which can be detected would be between V_min_ ≈ 0.4 and 4 mm^3^. This would be the minimum size of a tumor which could be detected with ICP-MS upon MSC homing with our Au NP approach. In summary, optimized Au NP labeling of MSCs and detection via ICP-MS appears suitable for in vivo tracking experiments.

#### In vivo tracking of Au NP labeled MSC

**Fig. 8:**
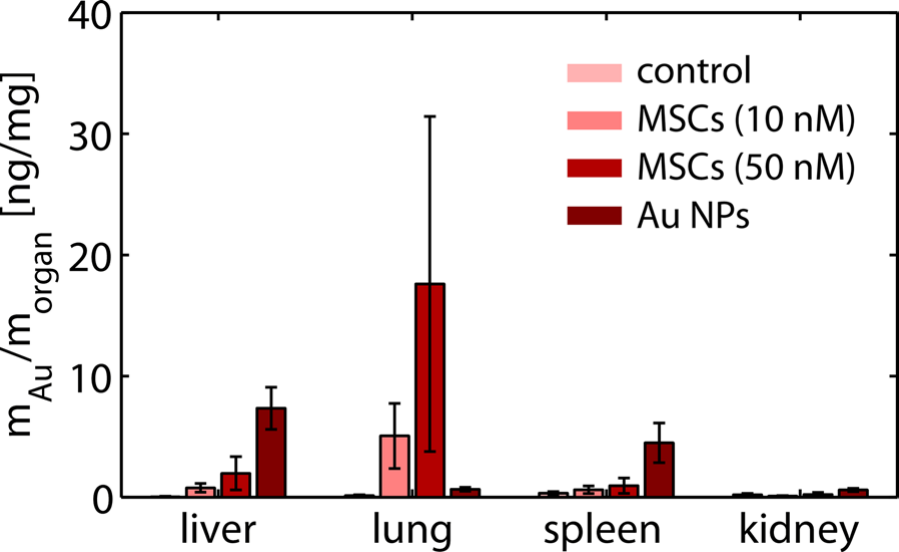
In vivo tissue distribution of MSCs labeled with Au NPs at concentrations of 10 and 50 nM for 48 h. Mice were injected in their tail vain with 50 μL of Au NP labeled MSCs (i.e. 10^6^ cells, which had been incubated with 10 or 50 nM Au NPs for 48 h). Alternatively, mice were injected in their tail vain with 50 μL of Au NPs at a concentration of 1300 nM. After 72 h, mice were sacrificed and the amount m_Au_ of Au in the different organs was determined with ICP-MS. The data show the mass of Au found per mass of organ from 5 independent experiments (n = 5)

As high concentrations of Au NPs in MSCs may lead to impaired homing efficiency, we sought to verify whether the migratory capacity of MSC was preserved after Au NP labeling in vivo. One million human MSCs were injected into the tail vain of two mice per condition, respectively. A solution of free Au NPs and phosphate buffered saline (PBS) served as control. 72 h after injection the mice were sacrificed and the amount of Au in liver, lung, spleen, kidney, and blood was determined via ICP-MS. For the control mice, the amount of detected Au was below 1 ppb and thus below the resolution, cf. Fig. 8. The injected free Au NPs, but not NP labeled MSCs, accumulated predominantly in the liver, as expected from previous studies with similar NPs [42, 43]. The difference was significant as calculated with Student’s *t* test (p = 0.005 and 0.04, respectively). In contrast, in mice injected with Au NP labeled MSCs a higher amount of Au was found inside the lungs. However, due to very high variations (p < 0.0001 upon F test for equality of variances) statistical significance was not reached here (p = 0.2). This is in agreement with findings by others, which have reported that MSCs get trapped in the pulmonary capillary system first, but then relocate into the liver or tumor/inflammation sites [44–46]. We conclude that in fact our optimized Au NP labeling protocol for human MSCs allows for proper recording the biodistribution of these cells. Note, that in fact some Au NPs associated to the MSCs could have been just adherent to the MSCs instead of being endocytosed. However, as the biodistribution of Au in case of Au NP labelled MSCs and plain Au NPs is different, the Au NPs must have travelled with the MSCs.

## Discussion

On the first glance, the physical properties of Au NPs for MSC labeling seem well suited when looking on the potential perspectives for further applications in cancer diagnostic and therapy. For clinical applications careful monitoring of cellular functions is a vital prerequisite. After thorough testing of biological alterations in NP labeled MSCs we propose an optimized labeling strategy for 4 nm Au NPs and human MSCs, hereby preserving migratory and proliferative capacities in vitro and in vivo. While in this study we demonstrated that exposure of MSCs to Au NPs at non-optimized conditions can have profound effects on the proliferation and migration behavior, the underlying molecular pathways that get disturbed so far are not known. Whether inorganic NP of different size, shape or material require the same or other labeling conditions with regard to MSC biology needs to be determined in further studies. We have previously employed short tandem-repeat (STR) profiling in order to quantify donor cells within recipient tissue. The sensitivity of this assay is about 5% [47]. Another strategy is fluorescent-dye based cell labeling. This method is capable to visualize MSC migration into tumors [48], however, quantification of light intensities in tissues is not always precise with regard to cell numbers. Thus, our approach of MSC quantification via Au NPs that is capable to detect 0.04% labeled MSCs within unstained cells is particularly accurate compared to the other mentioned methods.

We additionally show that there is a remarkable variety in-between individual donors, indicating the need to further elucidate the mechanisms of cellular fitness with regard to Au NP uptake capacity. MSCs administered intravenously initially migrate into the lungs, while intraarterial administration seems to prevent this ‘first-pass’ effect [44–46]. However, for photothermal tumor ablation intravenous application strategies for Au NP carrying MSCs are preferred [49]. Preservation of migratory capacities of MSC is therefore crucial for all these strategies.

## Conclusions

Tumor tropism of MSC has already been used for novel imaging approaches but also for cancer therapy strategies. With regard to the long hike throughout the body towards tumor tissue and considering future applications in cancer therapy, MSC fitness and migration capabilities appear to be of tremendous importance. We describe a gentle and efficient labeling strategy for human MSCs that is applicable in vivo and paves the way for future clinical applications such as novel tumor detection and destruction strategies.

## Methods

### Synthesis and characterization of NPs

Polymer-coated Au NPs with a core diameter of d_c_ = 4.25 ± 0.88 nm (as determined by transmission electron microscopy (TEM), in the following referred to as “d_c_ = 4 nm”), a hydrodynamic diameter of d_h_ = 10.4 ± 0.7 (as determined by dynamic light scattering (DLS) in water), and a zeta-potential of ζ = −25.1 ± 0.36 mV (as determined from laser doppler anemometry (LDA) in water) were prepared according to previously published protocols [30, 50]. The experimental procedure, as well as the effect of salt on the size and colloidal stability of PMA NPs, are described in detail in the Additional file 1. The NPs were overcoated with an amphiphilic polymer, poly(isobutylene-*alt*-maleic anhydride)-*graft*-dodecylamine (PMA) [51, 52]. After synthesis, the NPs were purified by gel electrophoresis and by diafiltration. The concentration of the coated Au NPs was determined by UV/Vis absorption spectroscopy [53]. For detailed characterization of the physicochemical properties of these NPs we refer to previous studies [20, 53–55]. The Au NPs were found to be colloidally stable up to physiological NaCl concentrations (see the Additional file 1).

### Isolation, expansion and culture of human mesenchymal stem cells (MSCs)

Mesenchymal stem cells were isolated from bone pieces obtained from hip fragments. Dulbecco’s Modified Eagles Medium (DMEM, Sigma-Aldrich, #D5546) was supplemented with 10% fetal bovine serum (FBS), 1% penicillin/streptomycin (P/S, Sigma-Aldrich, #P4333), and 2 mM l-glutamine (Sigma-Aldrich, #G7513). The MCS were cultivated in flasks at 37 °C and 5% CO_2_, until they reached 80% confluence. MSC where used in passages ≤5 due to observed adverse effects on MSC functional capabilities for higher passages as described previously [40].

### Quantification of Au NP uptake by MSCs

The labeling efficiency of MSCs with Au NPs (ca. 4 nm core diameter) was examined by inductively coupled plasma mass spectrometry (ICP-MS, Agilent 7700 Series). Cells were seeded into 6-well plates (TPP, Switzerland, #92006) at a density of 10^4^ MSCs/cm^2^ and each well with a surface of 9 cm^2^ was filled with V_medium_ = 3 mL of medium. Thus, in each well there were N_cell_ = 9·10^4^ cells. After 24 h, the growth medium was replaced with 1.5 mL of Au NP-containing media at different concentrations (c_NP_ = 0–100 nM) and cells were incubated for 5, 24, or 48 h. After exposure, the cell medium was removed followed by three washing steps with PBS to remove non-internalized NPs. Then, cells were detached with 500 µL trypsin–EDTA (0.05% trypsin–EDTA, Thermo Fisher Scientific), collected by centrifugation at 280 rcf for 5 min, and washed with PBS, followed by an additional centrifugation step. The recovered cell pellets were treated with 100 µL of lysis buffer (Luciferase Cell Culture Lysis Buffer, Promega, #E153A) for 30 min. Finally, the samples were prepared for ICP-MS analysis by digestion in aqua regia. Hereby, 50 µL sample was diluted in 150 µL aqua regia, consisting of three parts concentrated (35 wt%) HCl (Fisher Chemical, #7647010) and one part of (67 wt%) HNO_3_ (Fisher Chemical, #7697372), and left for digestion for at least 3 h. The sample containing acid was diluted 1:10 in 2 wt% HCl prior to measuring the elemental Au concentration in the sample with ICP-MS. The initial cell number was determined by performing a Lowry protein assay (Sigma-Aldrich, #TP0300) with the lysed cell pellets [56].

### Assessment of long term labeling efficiency of MSCs with Au NPs by reporting exocytosis versus endocytosis

For evaluation of the long-term labeling efficiency, the fraction of exocytosed NPs was determined after exposure to Au NPs. MSCs (adherent in 25 cm^2^ culture flasks) were exposed to c_NP_ = 2–100 nM of Au NPs for 24 or 48 h. After labeling, MSCs were detached with trypsin–EDTA, washed with PBS, and plated into new 25 cm^2^ culture flasks. After 24 or 48 h, the Au content remaining inside MSCs (i.e. the remaining endocytosed NPs) and present in growth medium (i.e. the exocytosed NPs) was determined. The intracellular Au was quantified by ICP-MS, as described above for the quantitative uptake analysis of Au NPs by MSCs. The exocytozed fraction of the Au NPs was determined from the Au concentration of the growth medium, which was diluted 1:4 in aqua regia first, followed by 1:10 dilution in 2 wt% HCl prior to ICP-MS measurements. Results are represented as Au mass fractions of intracellular versus the intracellular + extracellular Au.

### Viability of MSCs labeled with Au NPs

Mesenchymal stem cells were seeded at a density of 10^4^ cells/well into a 96-well plate with each well filled with V_medium_ = 0.1 mL of medium and exposed to Au NPs for 24–72 h. Then, cells were washed once with PBS and AlamarBlue (Thermo Fisher Scientific) was added in each well and incubated for desired time at 37 °C. The fluorescence was measured at 560 nm excitation and 590 nm emission wavelengths using a spectrophotometer (SpectraMax 250, Molecular Devices). Cell viability was assumed to be proportional to the recorded fluorescence intensity. Results are expressed as percentage of cell viability V versus control (i.e. untreated cells). Experiments were performed with MSCs from three independent human/MSC donors in triplicates for each time-point and concentration.

### Proliferation of MSCs

The effect of exposure of MSCs to Au NPs on their proliferation rate P was determined with carboxyfluorescein succinimidyl ester (CFSE) and flow cytometry (FCM). Cells were labeled with a certain amount of membrane-impermeable CFSE whose fluorescent intensity decreases upon cell division [57]. 2.5·10^5^ cells per sample were labeled with 1 µM CFSE (Molecular Probes, #C34554) for 10 min at 37 °C in 1 mL of PBS. Subsequently, the cells were washed twice with 5 mL of pre-warmed supplemented DMEM and plated in 25 cm^2^ culture flasks. After 24 h NPs (c_NP_ = 0–50 nM) were added, and a negative control was prepared containing 5 µM of the mitosis inhibitor cholchicine (Sigma-Aldrich, #C9754). After subsequent culturing for additional 6 days, cells were detached with trypsin–EDTA, counter-stained with 1 µM propidium iodide (PI, Sigma-Aldrich, #P4170), and signals were acquired with a BD LSR II FCM device with FACS Diva software (BD Biosciences). Data were analyzed with FlowJo version 9.5.3 (TreeStar Inc.) and GraphPad Prism software. CFSE was excited at 488 nm and emission was detected at 521 nm. Living cell were gated after 4′,6-diamidino-2-phenylindole (DAPI) (Sigma-Aldrich, #D9542) staining. Results are normalized to the positive (p = 1, no Au NPs) and negative control (p = 0, cholchicine), and are representing the mean values ± standard deviations of the median values of the CFSE fluorescent intensity/cell for 3 independent experiments.

### Migration of MSCs

The migration potential of MSCs was assessed by analyzing cell migration through membrane inserts by fluorescence microscopy [37]. MSCs were labeled with Au NPs in 25 cm^2^ culture flasks filled with 5 mL of medium at c_NP_ = 0–25 nM for 2 days. Subsequently, cells were detached with trypsin–EDTA and transferred in serum free medium into the upper chamber of membrane inserts (8 µm pore size, Greiner Bio One, #662638), which were placed into the wells of a 24 well plate (Greiner Bio One, #622160). Each insert contained 1·10^4^ cells in V_medium_ = 0.3 mL of growth medium. The lower chambers were filled with growth medium containing 10% humand platelet lysate (HPL, manufactured at the Institute for Clinical Immunology and Transfusion Medicine, Giessen, Germany, in a GMP-compliant manner as described in Schallmoser et al. [58]) to stimulate MSC migration from the upper to the bottom side of the membrane inserts. After 16 h, cells were fixed with methanol and nuclei were stained with 50 µM of 4′,6-diamidino-2-phenylindole (DAPI, Thermo Fisher Scientific, #D1306) for 5 min. For each sample, migrated and non-migrated MSCs were counted at fixed positions, each comprising an area of 0.38 cm^2^. The counting was based on fluorescent images acquired with a confocal laser scanning microscope (CLSM 510 Meta) from Zeiss using a Plan-Apochromat 20×/0.8 M27 objective (pinhole size: 1 airy unit, lateral sampling rate: 0.6 µm/pixel). DAPI (nuclei) was excited with a 405 nm-laser diode an emission was gated with a 420 nm long-pass filter. For imaging the inserts were placed on a microscope slide in a drop of PBS. For 4–6 randomly chosen positions two images were acquired: Non-migrated cells were captured by acquiring an image at a plane above the membrane, and migrated cells were imaged below the membrane, cf. the Additional file 1 for a sketch of the set-up. For each position (area A = 0.38 mm^2^) the number of cells above (N_non-mig_) and below the membrane (N_mig_) was determined based on their nuclear staining by employing CellProfiler [59] and the ratio N_mig_/(N_mig_ + N_non-mig_) was calculated. Results are displayed as mean values ± standard deviations for 3 independent experiments.

### Expression of surface markers of MSCs

The immunophenotype of MSCs was analyzed after exposure to 10 nM Au NPs for 48 h. According to the recommendations of the International Society for Cellular Therapy [38] the following surface markers were measured: CD14 (clone M4P9, BD Biosciences, #345785), CD19 (clone SJ25C1, BD, #332780), CD34 (clone 8G12, BD, #345801), CD45 (clone 2D1, BD, #332784), CD73 (clone AD2, BD, #550257), CD90 (clone 5E10, BD, #559869), CD105 (clone 266, BD, #32830) and HLA-DR (clone B8.12.2, Immunotech, #PNIM0463U). In brief, MSCs were stained for 15 min at 4 °C with fluorochrome-labeled monoclonal antibodies, washed with PBS, and resuspended in FACSFlow™ (BD, #342003) with 3% formaldehyde (Merck, #103999). The samples were measured with a LSRII FCM device with CellQuest Pro™ Software (both BD). Isotype-matched antibodies were used as negative controls (BD, #342409, #347221, #345818). FCS data were analyzed with FlowJo™ software version 9.5.3 (TreeStar Inc).

#### Sensitivity of MSC detection via ICP-MS

In order to prove dose dependency of our assay, dilutions of 10 nM Au NP labeled MSCs within HL-60 cells were prepared. 10^6^ of unlabeled HL-60 were diluted with 10–0.001% labeled MSCs in increments of 10 and measured via ICP-MS. Acute promyelocytic leukemia cells (HL-60) were obtained from the American Type Culture Collection (ATCC, Manassas, VA, USA), and were maintained in RPMI 1640 (Sigma-Aldrich, #R8758) supplemented with 10% FBS, 1% penicillin/streptomycin (P/S, Sigma-Aldrich, #P4333) (complete medium) at 37 °C in 5% CO_2_.

#### Demonstration of recording biodistributions with NP labeled MSCs

To evaluate the in vivo biodistribution of NP-labeled MSCs, male BALB/cAJic− RAG2−/− IL-2Rgamma−/− mice (obtained from Prof. Dr. Dorothee von Laer; Georg-Speyer-Haus; Johann Wolfgang Goethe-Universität Frankfurt) at 12–20 weeks of age were used. The experiments were performed in the animal facility of the BMFZ, Marburg, Germany. In brief, 1 × 10^6^ MSCs were seeded in T175 cm^2^ flasks and grown in complete cell culture medium. After 24 h, medium was replaced with NP containing media (10 and 50 nM Au NPs). MSC were incubated with Au NPs for 48 h to ensure cell labeling. After the desired time, the MCSs were washed three times with PBS, dissociated with trypsin, and resuspended in PBS. Subsequently, 50 μL of 1 × 10^6^ NPs labeled MSCs were injected in a tail vein of mice. Additionally, a group of Mice were injected with 50 μL of pure Au NPs at a concentration of 1300 nM. Mice injected with PBS were used as control. 72 h post injection, the mice were sacrificed, and the amount of Au in the lung, liver, spleen, kidney, and blood was evaluated by the ICP-MS. For the control mice group, the Au detected was below 1 ppb and thus below the resolution. Data shown represent an average of n = 5 independent experiments.

## Additional file

**Additional file 1.** Supplementary information about AuNP synthesis, purification, characteristics and about cell proliferation and migration assays.

## Abbreviations

MSCs: mesenchymal stromal cells
NP: nanoparticle
Au: gold
ICP-MS: inductively coupled plasma mass spectrometry
PET: positron or photon emission tomography
MRI: magnetic resonance imaging
FeO_x_: iron oxide
PBS: phosphate buffered saline
TEM: transmission electron microscopy
LDA: laser doppler anemometry
PMA: poly(isobutylene-*alt-*maleic anhydride)
CFSE: carboxyfluorescein succinimidyl ester
PI: propidium iodide
DAPI: 4′,6-diamidino-2-phenylindole
HL-60: human leukemia cell line
FBS: fetal bovine serum
