# Correction to: Optimizing conditions for labeling of mesenchymal stromal cells (MSCs) with gold nanoparticles: a prerequisite for in vivo tracking of MSCs

**DOI:** 10.1186/s12951-019-0527-6

**Published:** 2019-09-17

**Authors:** Philipp Nold, Raimo Hartmann, Neus Feliu, Karsten Kantner, Mahmoud Gamal, Beatriz Pelaz, Jonas Hühn, Xing Sun, Philipp Jungebluth, Pablo del Pino, Holger Hackstein, Paolo Macchiarini, Wolfgang J. Parak, Cornelia Brendel

**Affiliations:** 1 Department of Hematology, Oncology and Immunology, Philipps University Marburg, Marburg, Germany; 2 Department of Physics, Philipps-University of Marburg, Marburg, Germany; 3 Thoraxklinik at Heidelberg University Hospital, Heidelberg, Germany; 4 Institute for Clinical Immunology and Transfusion Medicine, Justus-Liebig University Giessen, Giessen, Germany; 5 Laboratory of Bioengineering & Regenerative Medicine (BioReM), Kazan Federal University, Kazan, Russia; 6 CIC Biomagune, San Sebastian, Spain

## Correction to: J Nanobiotechnol (2017) 15:24 10.1186/s12951-017-0258-5

Figure 5 was published incorrectly in the original publication of the article [1]. The correct version of Fig. 5 is given in this erratum.

**Fig. 5:**
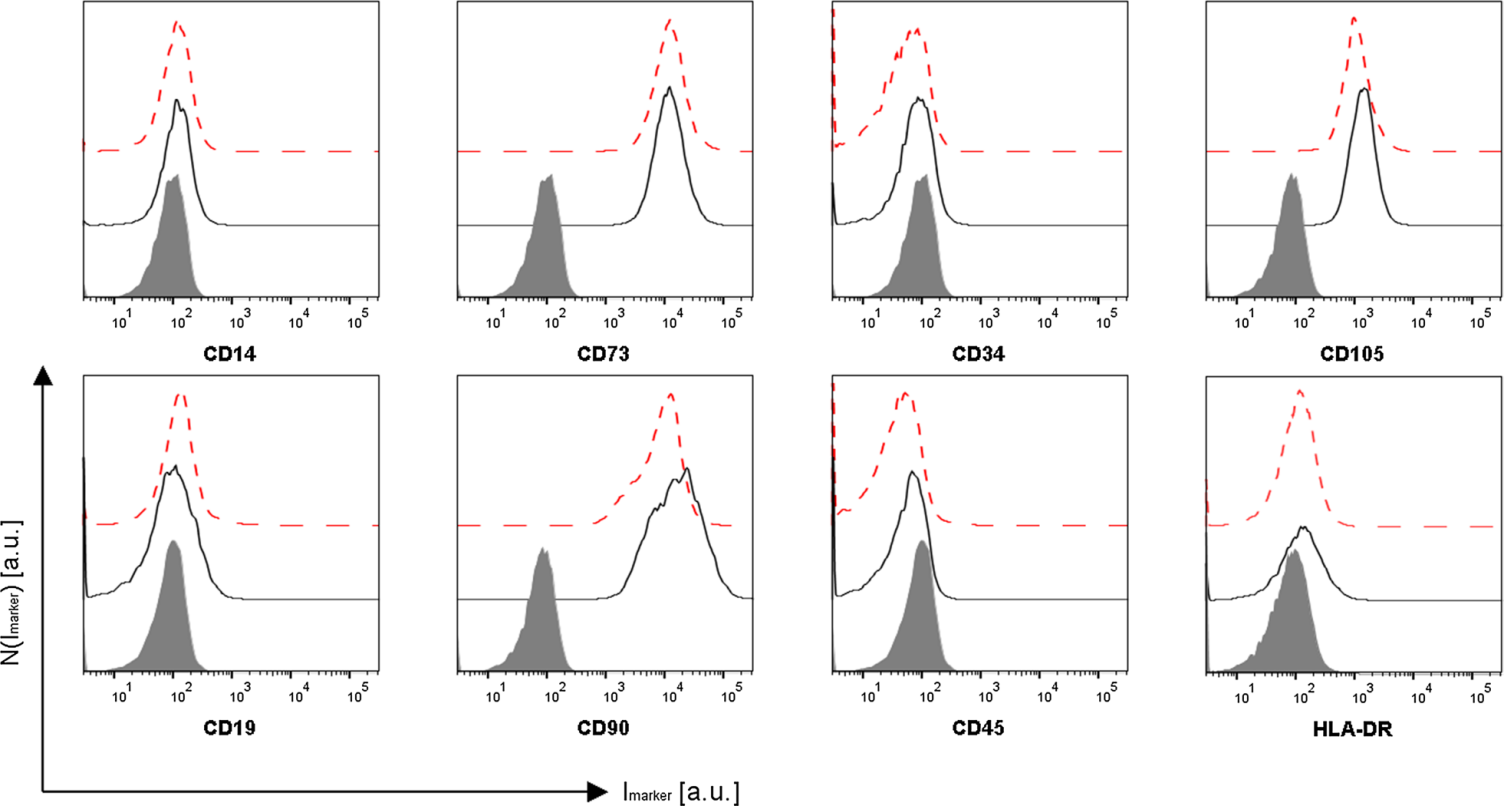
Internalized Au NPs do not affect surface marker expression of MSCs. Representative histograms of 3 independent experiments of the distribution of the marker fluorescence N(I_marker_) of MSC-defining surface markers of untreated MSCs (*black solid line*) and MSCs exposed to Au NPs at c_NP_ = 10 nM (*red dashed line*) for 48 h are shown. The solid grey front curve represents the isotype control

The authors apologize for the unfortunate error in the figure during publication of the article. It should also be clarified that some of the solid grey graphs in Fig. 5 are intentionally based on the same data. For 8 different surface makers (CD14, CD73, CD34, CD105, CD19, CD90, CD45, HA-DR) in accordance to the guidelines of the manufacturer a panel of 4 different isotype controls were used, corresponding to 4 different fluorescence channels.
