# Supplementary material for: Optimizing conditions for labeling of mesenchymal stromal cells (MSCs) with gold nanoparticles: a prerequisite for in vivo tracking of MSCs
Source: J Nanobiotechnology. 2017 Mar 29;15:24. doi: 10.1186/s12951-017-0258-5 (PMC5372278; doi:10.1186/s12951-017-0258-5)
Supplement: Supplementary file 1 — Additional file 1. Supplementary information about AuNP synthesis, purification, characteristics and about cell proliferation and migration assays. [file 12951_2017_258_MOESM1_ESM.docx]

**Optimizing conditions for labeling of mesenchymal stromal cells (MSCs) with gold nanoparticles – a prerequisite for *in vivo* tracking of MSCs**

Philipp Nold^1,#^, Raimo Hartmann^2,#^, Neus Feliu^2,^, Karsten Kantner^2^, Mahmoud Gamal^2^, Beatriz Pelaz^2^, Jonas Hühn^2^, Xing Sun^2^, Philipp Jungebluth^3^, Pablo del Pino^2^, Holger Hackstein^4^, Paolo Macchiarini^5^, Wolfgang J. Parak^2,6,*^, Cornelia Brendel^1,*^

^1^ Department of Hematology, Oncology and Immunology, Philipps University Marburg, Marburg, Germany.

^2^ Department of Physics, Philipps-University of Marburg, Marburg, Germany

^3^ Thoraxklinik at Heidelberg University Hospital, Heidelberg, Germany

^4^ Institute for Clinical Immunology and Transfusion Medicine, Justus-Liebig University Giessen, Giessen, Germany

^5^ Laboratory of Bioengineering & Regenerative Medicine (BioReM), Kazan Federal University, Russia

^6^ CIC Biomagune, San Sebastian, Spain

^#^ equally contributing first authors

^*^ corresponding authors: wolfgang.parak@physik.uni-marburg.de, brendelc@staff.uni-marburg.de

**SUPPLEMENTARY INFORMATION**

I) Synthesis of nanoparticles

II) Transfer of nanoparticles to water by polymer coating and purification

III) Characterization of nanoparticles

IV) Cell proliferation assay

V) Cell migration assay

VI) ICP-MS analysis

VII) Author contributions

VIII) References

**I) Synthesis of nanoparticles**

**I.1) Synthesis of 4 nm Au nanoparticles**

Synthesis was carried out following previously published protocols with some modifications [1, 2]. For all nanoparticle (NP) synthesis and characterization we also refer to a recently published protocol manuscript [3].

Phase transfer of precursor: 2.17 g of tetraoctylammonium bromide (TOAB, Sigma-Aldrich, #1643192) in 80 mL of toluene was firstly transferred into a 250 mL separation funnel, then 300 mg tetrachloroauric acid (HAuCl_4_, Strem Chemicals, #16903358) in 25 mL MilliQ-water was added into the funnel as well. After shaking for 5 min, the yellow solution in water was transferred into the colorless toluene, which means the Au^3+^ ions in aqueous phase were transferred into the organic phase. The aqueous phase was discarded and the toluene phase was transferred to a 250 mL round flask.

Reduction reaction for NPs nucleation and growth: Under vigorous stirring, 334 mg of sodium borohydride (Sigma-Aldrich, #71321) in 25 mL of water was added to the red solution within 1 min, and the color changed from red to red-violet. This color change indicates the nucleation of gold clusters mediated by sodium borohydride. The solution was kept stirring for 3 h. Afterward, the mixture was transferred to a cleaned separation funnel, and 25 mL of 10 mM HCl were added in order to remove the excess sodium borohydride. The funnel was shaken for 1 min, and the aqueous phase on the bottom was discarded. 25 mL of 10 mM of NaOH was added to the funnel to remove the excess acid, and after shaking for 1 min the aqueous phase was discarded again. Finally, 25 mL of water were added to remove excess ions, the funnel was shaken for 1 min and the aqueous phase was discarded. This last washing step was repeated 3 more times. The aqueous phase and the eventually remaining emulsion were discarded. The organic phase was then transferred to a 250 mL round flask and stirred over night to improve the NPs’ size distribution.

Ligand exchange: TOAB (in case of TOAB-coated Au NPs) is a relatively weak ligand compared with thiol-containing surfactants like dodecanethiol (DDT, Sigma-Aldrich, #112550), due to the high affinity of the thiol groups to the gold. For this reason, the Au NPs were subjected to a ligand exchange with DDT to enhance their colloidal stability. Briefly, 10 mL of dodecanethiol were added to the Au NPs dissolved in toluene. The solution was heated to 65 °C and stirred for 2 – 3 h. The solution was allowed to reach room temperature and the NPs were cleaned by precipitation to remove the substituent TOAB and unreacted DDT. First, the NPs were precipitated by the addition of ethanol and collected by centrifugation (5 min, 1000 g). The supernatant containing the free ligands was discharged, and the NPs were dispersed in chloroform and again cleaned twice by precipitation using the centrifugation (5 min, 1000 g) in order to remove big aggregates. Finally, the resulting NP solution was collected and the concentration of the NPs was determined by UV/Vis absorption spectroscopy (see §II.1).

**II) Transfer of nanoparticles to water by polymer coating and purification**

**II.1) Determination of nanoparticle concentrations**

**II.2) Polymer coating procedure**

**II.3) Purification of the nanoparticles**

**II.1) Determination of nanoparticle concentrations**

Two approaches were used. In the first approach the concentration of the Au nanoparticles c_NP_ was determined *via* UV/Vis absorption spectroscopy (Agilent 8453 spectrometer) by using the Beer-Lambert Law and the molar extinction coefficient (ɛ) [3]. The absorbance values measured at the wavelengths as indicated in Table (SI-II.1.):

| **Material** | **d_c_ [nm]** | **λ_ε_ [nm]** | **ε [M^-1^cm^-1^]** |
| --- | --- | --- | --- |
| Au | 4.25 ± 0.88 | 518 | 8.6 · 10^5^ [4] |

*Table SI-II.1. Value of the molar extinction coefficients ε used for the NPs sample. d_c_ refers to the (core) diameter of the NPs* *[5], and λ_c_ to the wavelength of the surface plasmon resonance peak.*

In the second approach the concentration of the Au NPs was derived from measurements of the elemental concentration of Au as determined by inductively coupled plasma mass spectrometry (ICP-MS) [3]. To determine the molar concentration of the Au NPs based on ICP-MS, the molar mass of M_NP(c)_ of one Au NP and the number of Au atoms per Au NP N_Au/NP_ were determined. The values for M_NP(c)_ and N_Au/NP_ for the Au NPs are shown below and have been calculated according to previous protocols [6]. Note, that M_NP(c)_ comprises only the mass of the Au core of the Au NP, and not the mass of the organic surface coating.

| **Material** | **d_c_ [nm]** | **M_NP(c)_ [g/mol]** | **N_Au/NP_** |
| --- | --- | --- | --- |
| Au | 4.25 ± 0.88 | 3.90 · 10^5^ | ≈ 3.86 · 10^3^ |

*Table SI-II.2. Values of the molar mass M_NP(c)_ and the number of Au atoms per NP N_Au/NP_ for the Au NPs.*

**II.2) Polymer coating procedure**

The NPs in chloroform were coated with the amphiphilic polymer poly (isobutylene–alt–maleic anhydride)–graft–dodecylamine (PMA, ~6,000 g/mol, Sigma-Aldrich, #531278) in order to transfer them to aqueous solution as reported by Lin *et al.* [2]. Briefly, 75% of the polymer anhydride rings were modified with dodecylamine (DDA, Sigma-Aldrich, #124221) in tetrahydrofuran (THF) at 65 °C under stirring. The polymer was dried using a Rotavapor under reduced pressure and dispersed in 20 mL of chloroform to produce a stock solution with final monomer concentration of 0.5 M.

The amount of PMA needed for coating of the NPs was calculated based on the total effective surface area (A_eff_) of the NPs, which was calculated using the equations reported by Soliman *et al*. [3, 7]. The NPs were mixed with a specific volume of PMA (monomer concentration c_p_ = 0.05 M) in a round flask and diluted with chloroform. Hereby, a determined amount of polymer was added depending on the NP surface area, given in terms of ratio R_p/Area_ of monomer units per nm^2^ of effective surface, see Table SI-II.3. After 30 min, the chloroform was slowly evaporated under reduced pressure using a Rotavapor at 45 °C in a bath, until the solvent was completely evaporated. After redispersion in chloroform the evaporation step was repeated twice. At the end the solid residue was dissolved in sodium borate (SBB pH = 12) and filtered through a syringe filter (0.22 μm pore size) in order to remove aggregated NPs.

| **Material** | **d_c_ [nm]** | R_p_/_Area_ [nm^-2^] | c_p_[M] |
| --- | --- | --- | --- |
| Au | 4.25 ± 0.88 | 350 | 0.05 |

*Table SI-II.3 Polymer coating for the NPs. c_p_ refers to the concentration of the polymer in stock solution in terms of monomer units, R_P/Area_ is the ratio of monomer per nm^2^ effective surface area needed to stabilize the NPs efficiently.*

**II.3) Purification of the nanoparticles**

The Au NPs (d_c_ = (4.25 ± 0.88) nm, see section 3.1) coated with PMA were cleaned using gel electrophoresis [8]. This was in particular necessary to remove empty polymer micelles from the solution. A 2% agarose gel was prepared by dissolving 3.5 g of agarose in 175 mL of Tris-borate-EDTA buffer (TBE 0.5x) and placing the solution into a gel tray. After 1 h, the gel was placed into the electrophoresis chamber filled with 0.5x TBE buffer. The sample containing the NPs was mixed with 10% v/v of loading buffer (30% glycerol and 0.3% Orange G in TBE 0.5x) to increase its density. After that, an electric field of 10 V·cm^-1^ was applied for 1 - 1.5 h. After migration of the negatively charged NPs to the anode, the gel was removed from the gel chamber, and the NPs band was cut out from the gel. The agarose piece containing the NPs was placed into a dialysis membrane (molecular weight cut-off (MWCO) = 50 kDa), and the NPs were extracted applying an electric field 10 V/cm for 20 min in the gel electrophoresis set-up. After that, the NPs were concentrated and the TBE 0.5 x was exchanged for water using centrifuge filters (*cf.* Table SI-II.2) for 3 times [8, 9].

**III) Characterization of nanoparticles**

**III.1) Transmission electron microscopy (TEM)**

**III.2) UV/vis absorption spectroscopy**

**III.3) Gel electrophoresis**

**III.4) Dynamic light scattering (DLS)**

**III.5) Laser Doppler anemometry (LDA)**

**III.6) The effect of salt on the colloidal stability of the NPs**

**III.1) Transmission electron microscopy (TEM)**

TEM was used to get information about the NPs’ size distribution, shape, and their colloidal stability. For TEM measurements, the samples were prepared by placing a drop of NP solution on top of a copper grid coated with a carbon layer. The size distribution was determined by counting more than 100 NPs in each TEM image using the free software ImageJ. The TEM picture and the corresponding size distribution histograms for the core diameters d_c_ of the NPs are presented in Figure SI-III.1

*Figure SI-III.1. TEM images of PMA-coated Au NPs dissolved in water with the respective histogram of their size distribution N(d_c_) of the inorganic core diameter d_c_. The scale bar corresponds to 100 nm. For the sample the following mean value ± standard deviation was obtained: d_c_ = 4.25 ± 0.88 nm.*

Note that in this study several batches of Au NPs were used. For the studies shown in §III.6 the bath of which the TEM size distribution is shown in Figure SI-III.2 was used. This also indicates the variability in size between different batches.

F*igure SI-III.2. TEM images of PMA-coated Au NPs dissolved in water with the respective histogram of their size distribution N(d_c_) of the inorganic core diameter d_c_. The scale bar corresponds to 50 nm. For the sample the following mean value ± standard deviation was obtained: d_c_ = 5.09 ± 0.65 nm.*

**III.2) UV/vis absorption spectroscopy**

UV/vis absorption spectra were recorded with an Agilent 8453 spectrometer. These measurements were used to measure the absorbance and to probe the stability of the NPs after polymer coating as shown in Figure SI-III.3.


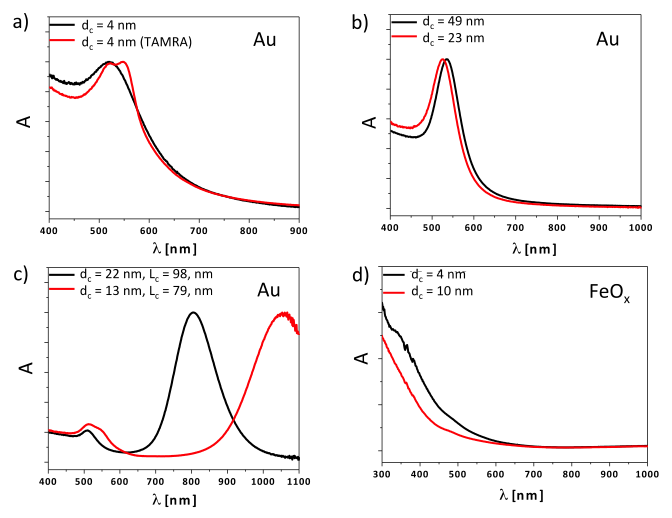


*Figure SI-III.3. Normalized UV/vis absorption spectra for PMA-coated Au NPs. In addition, a spectrum for Au NPs with the fluorophore Tamra (5-Carboxytetramethylrhodamine) [3] in the PMA shell is shown.*

**III.3) Gel electrophoresis**

The Au NPs were run on a 1 % Agarose gel at 10 V/cm for 1 h in Tris-Borate-EDTA buffer (TBE 0.5x). Au NPs of d_c_ = 10 nm stabilized with triphenyl phosphine (bis(p-sulfonatophenyl) phenylphosphine dehydrate were run in the gel as internal standard [10]. After PMA-coating, the Au NPs migrated through the gel towards the anode (+) due to their negative charge, *cf.* Figure SI-III.4. Migration in sharp bands is an indication for the colloidal stability of the NPs, which is due to the negative surface charge of carboxylic groups (-COO^-^) on the polymer surface.

*Figure SI-III.4. Pictures of gels on which PMA-coated NPs had been run after gel electrophoresis (1 h, 10 V/cm, 1% agarose gel) under white (left) and UV light excitation (right). The control sample is d_c_ = 10 nm triphenyl phosphine (bis(p-sulfonatophenyl) phenylphosphine dehydrate coated Au NPs. The “+” pole of the gel in on top of the image, the “-“ pole on the bottom.*

**III.4) Dynamic light scattering (DLS)**

The effective hydrodynamic diameter d_h_ (and thus also the colloidal stability; agglomerated NP show an increased hydrodynamic diameter [5]) of the NPs was measured with DLS (Malvern Zetasizer). The NPs sample were equilibrated for 10 min at 25 °C in order to ensure that the measured fluctuation signals belong to Brownian motion and not to any thermal conversion. The NPs were measured in water at 173° backscatter settings, using a 633 nm laser. The determined hydrodynamic diameter d_h_ is shown in Figure SI-III.5 and in Table SI-III.1

*Figure SI-III.5. Number distribution of the hydrodynamic diameters N(d_h_) for PMA-coated NPs dissolved in water.*

| **d_c_ [nm]** | **L_c_ [nm]** | **d_h_ [nm]**  **number distribion** | **Z_ave_ [nm]** | **PDI** | **ζ [mV]** |
| --- | --- | --- | --- | --- | --- |
| 4.25 ± 0.88 | - | 10.4 ± 0.7 | 47.7 ± 0.5 | 0.59 ±0.01 | - 25.1 ± 0.36 |

*Table SI-III.1. Mean value ± standard deviation of the hydrodynamic diameter d_h_ of the Au NPs dissolved in water as derived from the number N(d_h_) distribution by DLS. Also Z-average (Z_ave_), polydispersity index (PDI), and ζ -potential values are listed.*

**III.5) Laser Doppler anemometry (LDA)**

The ζ-potential of the NPs was measured with the same set-up as used for DLS. The resulting data are given in Figure SI-III.6, confirming the negative charge of the NPs

F*igure SI-III.6. ζ****-****potential distribution N(ζ) for PMA-coated NPs dissolved in water.*

**III.6 ) The effect of salt on the colloidal stability of the NPs**

To study salt-induced aggregation of the as-synthesized Au NPs, dynamic light scattering (DLS) was used to analyze the behavior of the Au NPs in increasing concentrations of NaCl [3]. For that, a new batch of Au NPs was synthetized as previously described in §III.1. The TEM images and size distribution of the synthetized NPs are shown in Figure SI-III.7. To evaluate the effect of salt on the colloidal stability, 0.5 mL of Au NP stock solution (50 nM) were mixed with 0.5 mL aq. NaCl solution of 0.01 - 5 M. In this manner, the concentration of Au NPs as well as for NaCl was cut by half leading to concentrations of c_NP_ = 25 nM and c_NaCl_ = 0.005 - 2.5 M. The hydrodynamic diameter was determined right after the mixing at t = 0 h and after an incubation time over night at t = 24 h (Figure SI-III.7) [3]. The row data are represented in Figure SI-III.8. Data indicate good colloidal stability at physiological NaCl concentrations (c_NaCl_ = 150 mM) [3].

*
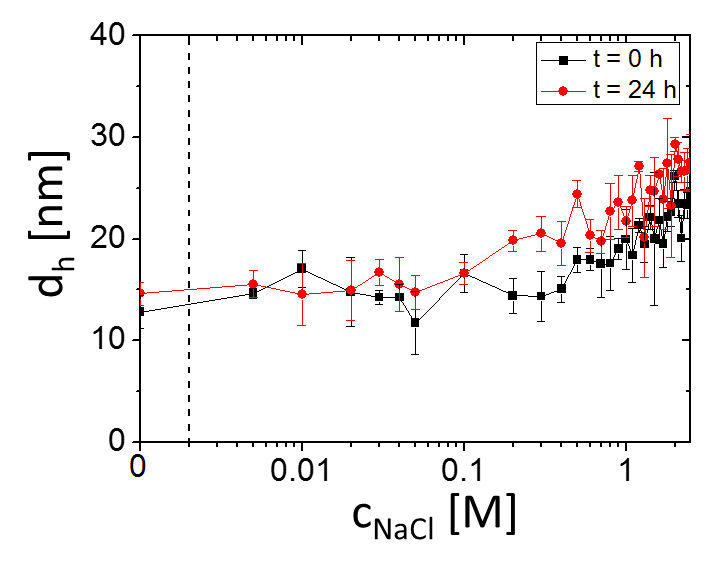
*

*Figure SI-III.7. The effect of salt on the colloidal stability of PMA coated Au NPs. A suspension of c_NP_ = 50 nM in milliQ was mixed in a volume ratio of 50:50 with aq. NaCl solutions (c_NaCl_ = 0.01 – 5 M) to reach final concentrations of c_Au_ = 25 nM and c_NaCl_ = 0.005 – 2.5 M. The hydrodynamic diameter d_h_ derived from the DLS number distribution was determined at t = 0 h and t = 24 h. Each data point consists of 3 consecutive measurements obtained for 10 run intervals with a run time of 5 s.*


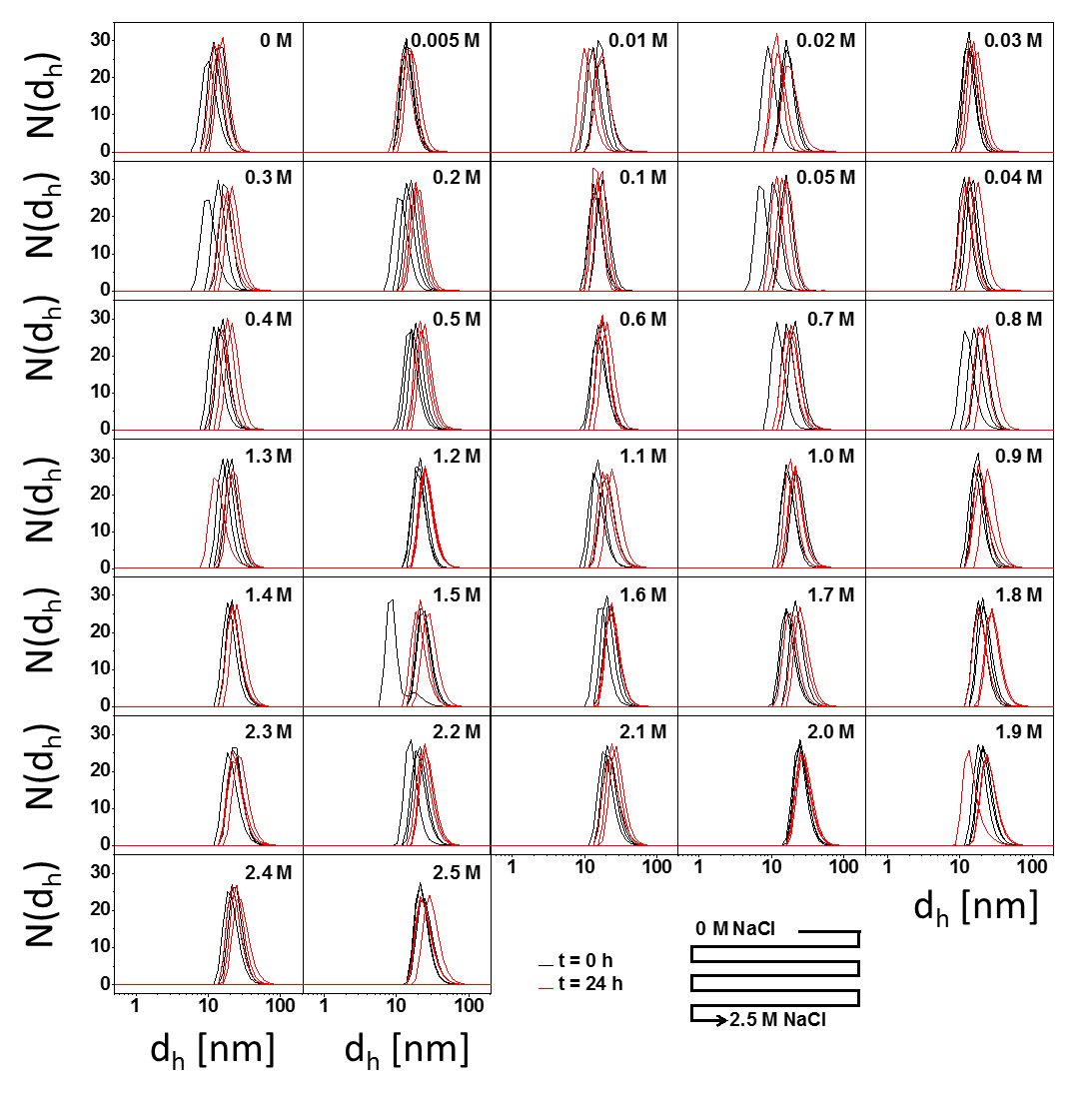


F*igure SI-III.8*. The effect of salt on the colloidal stability of PMA-coated Au NPs. Representation of the raw data (number distributions) used for the evaluation shown in Figure SI-III.7 [3].

**IV) Cell proliferation assay**

**IV.1) Flow cytometry measurements**

**IV.2) Fluorescence microscopy measurements**

**IV.1) Flow cytometry measurements**

**
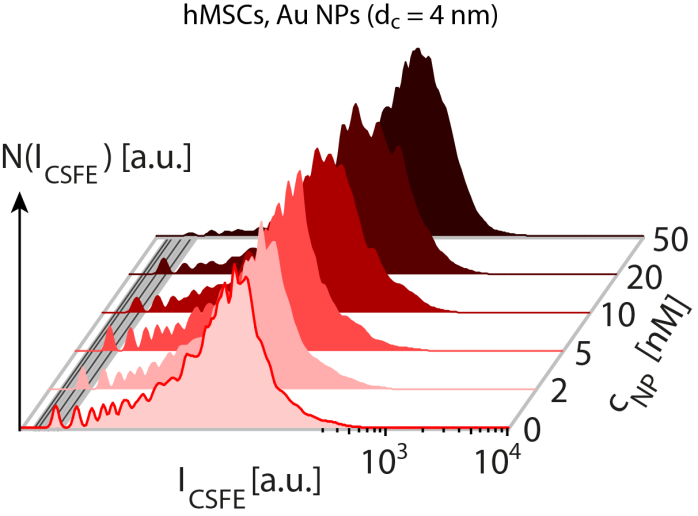
**

Figure SI-IV.1. Raw data for the proliferation assays as presented in the main manuscript. The distributions N(I_CSFE_) of CFSE intensities I_CSFE_ per cell after exposure to the indicated concentrations for 1 week are shown. A low fluorescence intensity of CFSE indicates proliferation, while an increase reflects a decreased proliferation rate.

**IV.2) Fluorescence microscopy measurements**

In addition, cellular proliferation was investigated by measuring the incorporation of the thymidine-analogue EdU (5-ethynyl-2 ́-deoxyuridine) during DNA synthesis. EdU can be detected by a copper-catalyzed click-reaction between its alkyne group and an azide group-containing fluorescent dye [11]. Cells were seeded into 96-well plates (6000 cells/well) and were exposed to NPs in complete medium at indicated concentrations for 24 h. The mitosis inhibitor colchicine (Sigma-Aldrich, #C3915) was used as negative control. Afterwards, the growth medium was replaced with medium containing 5 µM EdU (Thermo Fisher Scientific, #A10044). After additional 6 h, the cells were fixed with methanol and incorporated EdU was labeled with AlexaFluor 488 azide. Therefore, cells were washed twice after fixation and treated with 280 mM Tris buffer (pH 8.5, Fisher Scientific, #BP152-1) containing 820 µM CuSO_4_ (Sigma-Aldrich, #61230), 30 µM AlexaFluor 488 azide (Thermo Fisher Scientific, #A10266), and 80 mM ascorbic acid (Sigma-Aldrich, #255564) for 30 min. Finally, cells were stained with 4’,6-diamidino-2-phenylindole (DAPI), and images were taken using an Axiovert 200M fluorescence widefield microscope (Zeiss). EdU-AlexaFluor 488 was exited at λ_ex_ = 480 nm (30 nm width band-pass filter) and emission was recorded at λ_em_ = 535 nm (40 nm width band-pass filter: green channel). Hoechst 33342 was excited around λ_ex_ = 365 nm and the emission signal was measured at λ_em_ = 440 nm (20 nm width band-pass filter: blue channel). Images of the whole 96-well plate were automatically acquired, using the open source software µManager and the HCS module [12]. Per well a total area of 20 mm² was screened, allowing for the analysis of almost all cells present in each well. For determining the proliferation rate p the images were processed using Matlab (Mathworks) and CellProfiler [13]. First, the DAPI-stained nuclei were segmented, representing all cells (proliferated and non-proliferated) present in the current image. Second, the mean fluorescence intensity in the blue channel (I_DAPI_) per nuclei was determined, as well as the mean intensity of EdU-AlexaFlour 488 in the corresponding green channel (I_EdU-AF488_). For each nucleus I_Dapi_ was plotted *versus* log(I_EdU-AF488_) in a scatter plot to identify the different cell populations (Figure SI-IV.1). Finally the fraction p of proliferated cells was calculated for each concentration of NPs.


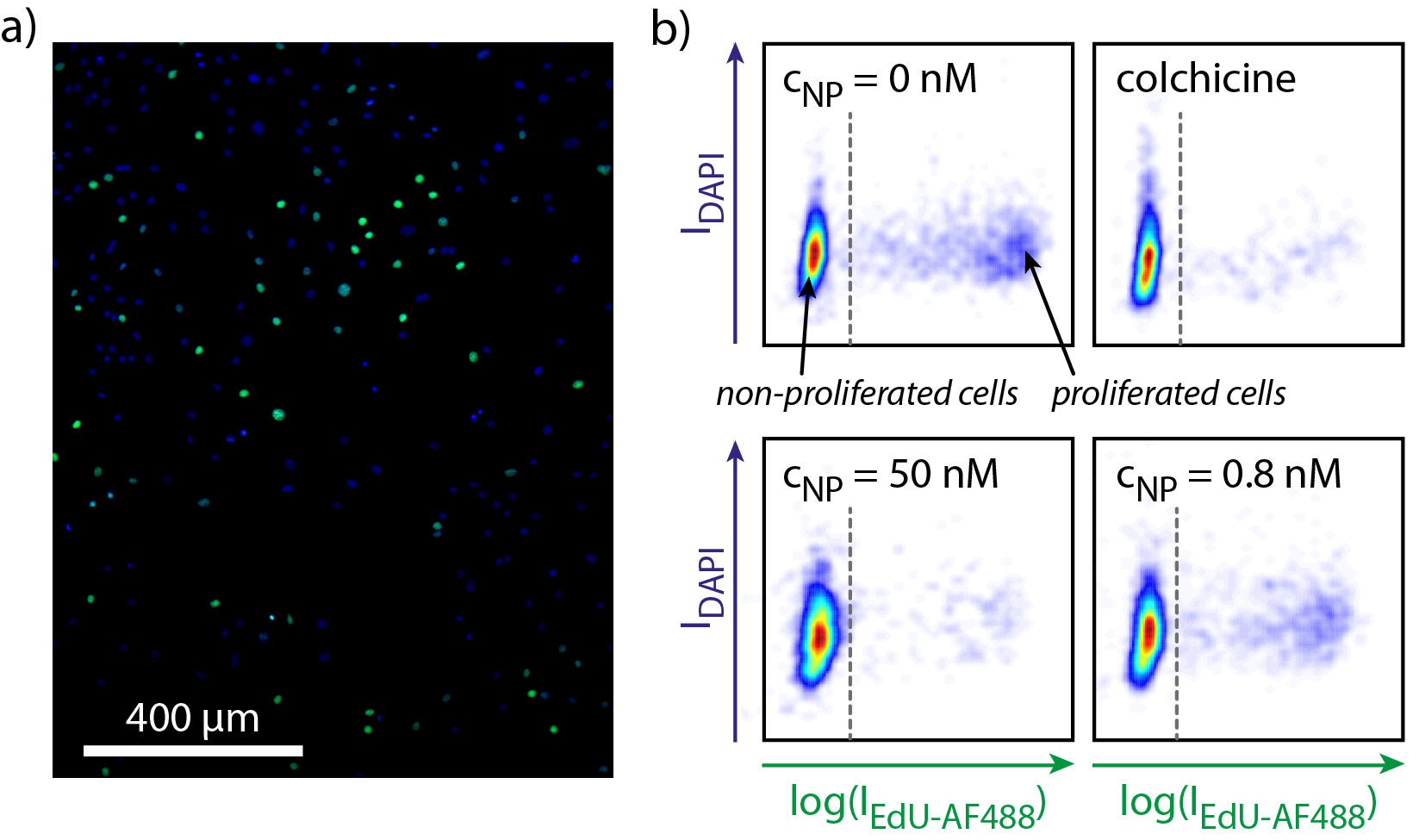


*Figure SI-IV.1. Cellular proliferation as measured by the incorporation of the thymidine-analogue EdU during DNA synthesis. a) Micrographs showing the fluorescence of DAPI (blue) and AF488-labeld EdU (green). b) Scatter plots displaying the mean intensity along each nucleus in the green intensity (I_EdU-AF488_) versus the corresponding mean intensity in the blue channel (I_DAPI_) at indicated conditions.*

**V) Cell migration assay**

**
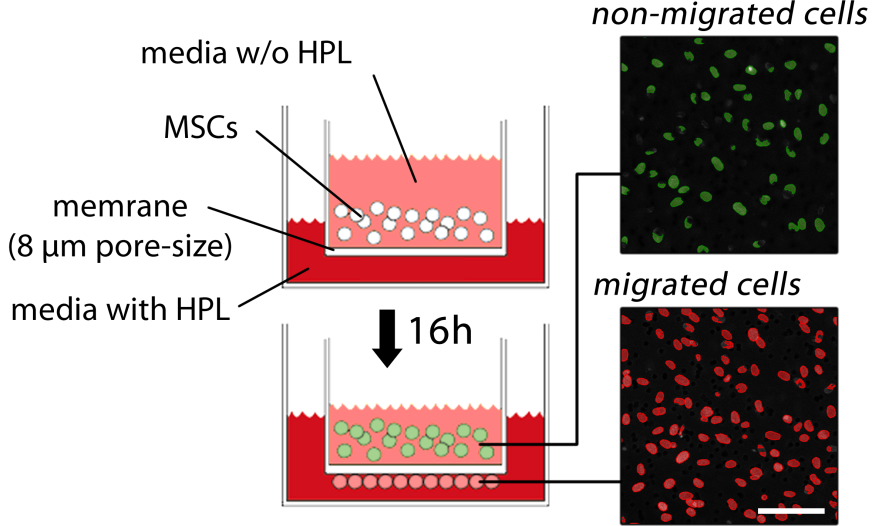
**

*Figure SI-V.1. MSC migration assay. hMSCs migrate from the upper to the lower chamber driven by the presence of human platelet lysate (HPL) in the growth medium filled into the lower chamber. For investigation of the migration nuclei of the respective cells were imaged at a certain position on top and below the membrane by confocal fluorescence microscopy. Then, objects were segmented and counted by employing CellProfiler [13] (identified nuclei are rendered exemplary in red and green). The scale bar corresponds to 100 µm.*

**VI) ICP-MS analysis**

ICP-MS analysis was carried out to determine the amount of Au found in the different organs of mice after intravenous injection of Au NPs similar to previous reports [6, 14]. The extracted mouse organs with a weighted mass of m_Organ_ were dissolved prior to measurement using 67 wt% HNO_3_ with a volume V_HNO3_ = 5 mL (Fisher Chemical, #7697-37-2) for 48 hours under constant agitation at room temperature. Note that in a previous study V_HNO3_ = 2 mL were used instead [6]. From the solution containing the digested organ in HNO_3_, 200 µL were taken for analysis, mixed with 400 µL for mice #5-#8 or 300 µL for mice #9-#12 of 37 wt% HCl (Fisher Chemical, #7647-01-0), *i.e.* dilution by a factor of 3 or 2.5, in order to enhance the digestion of the incorporated Au NPs. This solution was after 2 hours again diluted by a factor of 10 with 2 wt% HCl to protect the ICP-MS setup, resulting in an overall dilution factor α_dil_ = 30 or 25. This diluted solution was then measured 5 consecutive times to determine the mass-concentration C'_Au_ of elemental gold in solution. C'_Au_ [µg/L] describes the solutions concentration. µg/L is also referred to as ppb (parts per billion or 1 µg/kg = 10^-9^ g per g), as the density of all used solutions is equal to the one of water = 1 kg/L. ΔC'_Au_ [%] describes the mean deviation between the 5 measurements. Thus the concentration in the original solution with the digested organ was calculated to C_Au_ [ppb] = C'_Au_⋅α_dil_. The total mass of gold per organ was calculated as m_Au_ [g] = C_Au_⋅V_HNO3_. Thus, the mass of gold per mass of organ is m_Au_/m_Organ_ = m_Au_/m_Organ_. In a previous work [6] the results were presented as C_Au_/m_Organ_ [ppb/g], whereby the scaling factor to m_Au_/m_Organ_ is V_HNO3_. In the following table all relevant ICP-MS data used in this study are listed, including the raw data. As example: liver of mouse #5: C'_Au_ = 73.57 µg/L → C_Au_ = C'_Au_⋅α_dil_ = 73.57 µg/L ⋅ 30 = 2207 µg/L → m_Au_ = C_Au_⋅V_HNO3_ = 2207 µg/L ⋅0.005 L = 11035.5 ng → m_Au_/m_Organ_ = 11035 ng/1520 mg = 7.26 ng/mg, and C_Au_/m_Organ_ = 2207 ppb / 1520 mg = 1.45 ppb/mg

| # mouse | investi-gated organ | C'_Au_ [ppb] | ΔC'_Au_ [%] | m_Organ_ [mg] | m_Au_/m_Organ_ [ng/mg] | C_Au_/m_Organ_ [ppb/mg] |
| --- | --- | --- | --- | --- | --- | --- |
| 5 | liver | 73.57 | 10.13 | 1520 | 7.260 | 1.45 |
| 5 | lung | 157.46 | 9.62 | 330 | 71.573 | 14.31 |
| 5 | tail | 2.38 | 1.45 | 270.3 | 1.321 | 0.26 |
| 5 | spleen | 5.22 | 3.82 | 242.3 | 3.234 | 0.65 |
| 5 | kidney | 2.81 | 5.25 | 484 | 0.872 | 0.17 |
| 6 | liver | 120.93 | 10.56 | 1735 | 10.455 | 2.09 |
| 6 | lung | 2.15 | 7.06 | 262.3 | 1.231 | 0.25 |
| 6 | tail | 70.15 | 10.09 | 540.5 | 19.467 | 3.89 |
| 6 | spleen | 2.63 | 4.22 | 36.9 | 10.700 | 2.14 |
| 6 | kidney | 1.63 | 2.44 | 609.2 | 0.402 | 0.08 |
| 7 | liver | 12.45 | 2.15 | 1519.3 | 1.23 | 0.25 |
| 7 | lung | 20.09 | 5.18 | 245.3 | 12.28 | 2.46 |
| 7 | tail | 0.71 | 10.57 | 252.5 | 0.42 | 0.08 |
| 7 | spleen | 1.61 | 3.00 | 219 | 1.10 | 0.22 |
| 7 | kidney | 0.74 | 5.18 | 524.5 | 0.21 | 0.04 |
| 8 | liver | 0.07 | 24.47 | 1928.4 | 0.01 | 0.00 |
| 8 | lung | 0.35 | 6.78 | 336.5 | 0.16 | 0.03 |
| 8 | tail | 0.12 | 25.46 | 236.7 | 0.08 | 0.02 |
| 8 | spleen | 0.13 | 20.19 | 105.6 | 0.19 | 0.04 |
| 8 | kidney | 0.25 | 15.33 | 520.5 | 0.07 | 0.01 |
| 8 | liver | 1.13 | 3.33 | 1090.4 | 0.13 | 0.03 |
| 9 | lung | 0.80 | 42.39 | 263.9 | 0.38 | 0.08 |
| 9 | tail | 2.47 | 14.65 | 232.9 | 1.33 | 0.27 |
| 9 | spleen | 0.80 | 9.20 | 134.4 | 0.74 | 0.15 |
| 9 | kidney | 1.78 | 28.38 | 334.6 | 0.66 | 0.13 |
| 10 | liver | 30.87 | 2.99 | 1095.2 | 3.52 | 0.70 |
| 10 | lung | 0.71 | 6.56 | 237.7 | 0.38 | 0.08 |
| 10 | tail | 38.98 | 2.11 | 770.2 | 6.33 | 1.27 |
| 10 | spleen | 0.64 | 1.56 | 86.5 | 0.93 | 0.19 |
| 10 | kidney | 0.99 | 2.06 | 287.6 | 0.43 | 0.09 |
| 11 | liver | 13.98 | 4.29 | 1032.8 | 1.69 | 0.34 |
| 11 | lung | 18.81 | 11.26 | 305.7 | 7.69 | 1.54 |
| 11 | tail | 1.51 | 3.74 | 113.7 | 1.66 | 0.33 |
| 11 | spleen | 0.70 | 4.13 | 65.7 | 1.34 | 0.27 |
| 11 | kidney | 0.24 | 2.50 | 320.5 | 0.10 | 0.02 |
| 12 | liver | 24.30 | 1.20 | 1491.9 | 2.04 | 0.41 |
| 12 | lung | 28.81 | 6.35 | 227.4 | 15.84 | 3.17 |
| 12 | tail | 36.11 | 3.03 | 260.2 | 17.35 | 3.47 |
| 12 | spleen | 0.55 | 2.50 | 48.9 | 1.41 | 0.28 |
| 12 | kidney | 0.63 | 1.45 | 533.2 | 0.15 | 0.03 |

*Table SI-VI.1: ICP-MS data of all organ samples from treated mice. Calculations were done as described above with V_HNO3_* *= 0.005 L and* α_dil_ *= 30 (mice #5-#8) and* α_dil_ *= 25 (mice #9-#12).*

**VII) Author contributions**

This work was started in 2013 and the revision was finished in 2017. All experimental work was carried out at the Philipps University Marburg. Neus Feliu had had an initial training in the group of Philipp Jungebluth and Paolo Macchiarini, with whom this project also had been discussed in 2014. Her experimental work regarding this study was carried out as guest researcher exclusively in the group of Wolfgang Parak, together with the group of Cornelia Brendel.

- Philipp Nold: prepared the stem cells and labelled them with NPs, carried out the cell migration assays, performed the in vivo experiments, carried out part of the evaluation.
- Raimo Hartmann: carried out parts of the viability, migration and proliferation assays, carried out part of the evaluation.
- Neus Feliu: carried out part of the viability assays, carried out part of the evaluation, supervised viability assays.
- Karsten Kantner: carried out ICP-MS measurements and evaluations, and prepared part of the Au NPs.
- Mahmoud Gamal: prepared part of the Au NPs.
- Beatriz Pelaz: supervised the synthesis of Au NP preparation.
- Jonas Hühn: prepared part of the Au NPs and carried out NP characterization.
- Xing Sun: carried out part of the viability assays.
- Philipp Jungebluth: consulted part of the initial uptake studies.
- Pablo del Pino: supervised the NP characterization.
- Holger Hackstein: consulted in vivo work.
- Paolo Macchiarini: consulted part of the initial uptake studies.
- Wolfgang J. Parak: conceived the concept of this work, designed experiments and co-wrote the manuscript.
- Cornelia Brendel: conceived the concept of this work, designed experiments, responsible for all animal experiments, and co-wrote the manuscript.

**VIII) References**

1. Brust M, Walker M, Bethell D, Schiffrin DJ, Whyman R: Synthesis of Thiol-Derivatized Gold Nanoparticles in a 2-Phase Liquid-Liquid System. *J Chem Soc, Chem Commun* 1994, 1:801-802.

2. Lin C-AJ, Sperling RA, Li JK, Yang T-Y, Li P-Y, Zanella M, Chang WH, Parak WJ: Design of an Amphiphilic Polymer for Nanoparticle Coating and Functionalization. *Small* 2008, 4:334-341.

3. Hühn J, Carrillo-Carrion C, Soliman MG, Pfeiffer C, Valdeperez D, Masood A, Chakraborty I, Zhu L, Gallego M, Zhao Y, et al: Selected Standard Protocols for the Synthesis, Phase Transfer, and Characterization of Inorganic Colloidal Nanoparticles. *Chemistry of Materials* 2017, 29:399−461.

4. Hühn D, Kantner K, Geidel C, Brandholt S, De Cock I, Soenen SJH, Rivera Gil P, Montenegro J-M, Braeckmans K, Müllen K, et al: Polymer-Coated Nanoparticles Interacting with Proteins and Cells: Focusing on the Sign of the Net Charge. *ACS Nano* 2013, 7:3253-3263.

5. Rivera Gil P, Jimenez de Aberasturi D, Wulf V, Pelaz B, del Pino P, Zhao Y, de la Fuente J, Ruiz de Larramendi I, Rojo T, Liang X-J, Parak WJ: The Challenge to Relate the Physicochemical Properties of Colloidal Nanoparticles to Their Cytotoxicity. *Accounts of Chemical Research* 2013, 46:743-749.

6. Kreyling WG, Abdelmonem AM, Ali Z, Alves F, Geiser M, Haberl N, Hartmann R, Hirn S, de Aberasturi DJ, Kantner K, et al: In vivo integrity of polymer-coated gold nanoparticles. *Nat Nanotechnol* 2015, 10:619-623.

7. Soliman MG, B BP, Parak WJ, Pino Pd: Phase transfer and polymer coating methods toward improving the stability of metallic nanoparticles for biological applications. *Chemistry of Materials* 2015, 27:990-997.

8. Fernández-Argüelles MT, Yakovlev A, Sperling RA, Luccardini C, Gaillard S, Medel AS, Mallet J-M, Brochon J-C, Feltz A, Oheim M, Parak WJ: Synthesis and Characterization of Polymer-Coated Quantum Dots with Integrated Acceptor Dyes as FRET-based Nanoprobes. *Nano Letters* 2007, 7:2613-2617.

9. Zhang F, Lees E, Amin F, Rivera_Gil P, Yang F, Mulvaney P, Parak WJ: Polymer-Coated Nanoparticles: A Universal Tool for Biolabelling Experiments. *Small* 2011, 7:3113-3127.

10. Pellegrino T, Sperling RA, Alivisatos AP, Parak WJ: Gelelectrophoresis of Gold-DNA Nanoconjugates. *J Biomed Biotechnol* 2007, Article ID 26796:1-9.

11. Salic A, Mitchison TJ: A chemical method for fast and sensitive detection of DNA synthesis in vivo. *Proceedings of the National Academy of Sciences* 2008, 105:2415-2420.

12. Edelstein AD, Tsuchida MA, Amodaj N, Pinkard H, Vale RD, Stuurman N: Advanced methods of microscope control using µManager software. *2014* 2014.

13. Carpenter A, Jones T, Lamprecht M, Clarke C, Kang I, Friman O, Guertin D, Chang J, Lindquist R, Moffat J, et al: CellProfiler: image analysis software for identifying and quantifying cell phenotypes. *Genome Biology* 2006, 7:R100.

14. Colombo M, Fiandra L, Alessio G, Mazzucchelli S, Nebuloni M, Palma CD, Kantner K, Pelaz B, Rotem R, Corsi F, et al: Tumour homing and therapeutic effect of colloidal nanoparticles depend on the number of attached antibodies. *Nature Communications* 2016, 7:13818.
